# Long noncoding RNAs with peptide‐encoding potential identified in esophageal squamous cell carcinoma: *KDM4A‐AS1*‐encoded peptide weakens cancer cell viability and migratory capacity

**DOI:** 10.1002/1878-0261.13424

**Published:** 2023-04-10

**Authors:** Bo Zhou, Yuyang Wu, Pei Cheng, Chengyong Wu

**Affiliations:** ^1^ Medical Research Center & Institute of Digestive Disease The Second Affiliated Hospital of Zhengzhou University China; ^2^ Scientific Research and Foreign Affairs Office The Second Affiliated Hospital of Zhengzhou University China; ^3^ Department of Urology The Second Affiliated Hospital of Zhengzhou University China; ^4^ Academy of Medical Sciences Zhengzhou University China

**Keywords:** esophageal squamous cell carcinoma, *KDM4A‐AS1*, lncRNAs, mass spectrometry, peptide

## Abstract

Currently, the knowledge of long noncoding RNA (lncRNA)‐encoded peptides is quite lacking in esophageal squamous cell carcinoma (ESCC). In this study, we simultaneously identified six lncRNA open reading frames (ORFs) with peptide‐coding abilities including lysine‐specific demethylase 4A antisense RNA 1 (*KDM4A‐AS1*) ORF by combining weighted gene co‐expression network analysis (WGCNA) for ESCC clinical samples, ribosome footprints, ORF prediction, mass spectrometry (MS) identification, and western blotting. *KDM4A‐AS1* ORF‐encoded peptide reduced ESCC cell viability and migratory ability. Co‐immunoprecipitation and MS analysis revealed that *KDM4A‐AS1*‐encoded peptide specifically bound with 103 proteins in ESCC cells, and enrichment analysis suggested that peptide‐bound proteins were related to fatty acid metabolism and redox process. Cell and molecular experiments demonstrated that *KDM4A‐AS1*‐encoded peptide inhibited stearoyl‐CoA desaturase and fatty acid synthase expression, increased reactive oxygen species level, and reduced mitochondrial membrane potential in ESCC cells. In summary, multiple lncRNAs with translation potential were simultaneously identified by combining multiple approaches in ESCC, providing novel identification strategies for lncRNA‐encoded peptides. Moreover, lncRNA *KDM4A‐AS1*‐encoded peptide weakened ESCC cell viability and migratory capacity and functioned in fatty acid metabolism and redox process.

## Graphical abstract

In this text, six lncRNA open reading frames (ORFs) with peptide‐coding abilities including KDM4A‐AS1 ORF were simultaneously identified by combing WGCNA, ribosome footprint datasets, ORF prediction, mass spectrometry identification, and western blotting in ESCC. KDM4A‐AS1 ORF‐encoded peptide reduced ESCC cell viability and migratory ability, inhibited fatty acid metabolism, and enhanced oxidative stress.

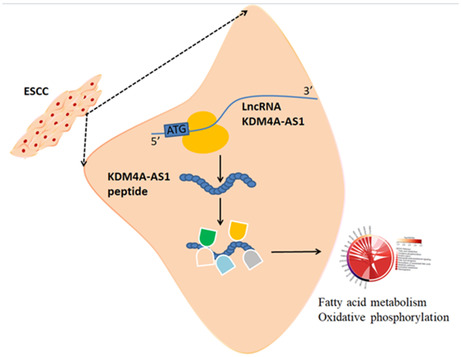

**Abbreviations**
ACOX1: peroxisomal acyl‐coenzyme A oxidase 1
ANOVA: one‐way analysis of variance
ASAP: ATP synthase‐associated peptide
AUC: area under the curve
CCK‐8: Cell Counting Kit‐8
cDNA: complementary DNA
Co‐IP: co‐immunoprecipitation
DDX11‐AS1: DDX11 antisense RNA 1
DLX6‐AS1: DLX6 antisense RNA 1
EC: esophageal cancer
ESCC: esophageal squamous cell carcinoma
FASN: fatty acid synthase
FBS: fetal bovine serum
IRES: internal ribosome entry site
KDM4A‐AS1: lysine‐specific demethylase 4A antisense RNA 1
KMT2E‐AS1: KMT2E antisense RNA 1
KTN1‐AS1: KTN1 antisense RNA 1
LINC00665: long intergenic non‐protein‐coding RNA 665
LINC00839: long intergenic non‐protein‐coding RNA 839
LINC01116: long intergenic non‐protein‐coding RNA 1116
LMO7‐AS1: LMO7 antisense RNA 1
LncRNA: long noncoding RNA
LOXL1‐AS1: lysyl oxidase homolog 1 antisense RNA 1
MMP: mitochondrial membrane potential
MS: mass spectrometry
ORF: open reading frame
Pep‐DDX11‐AS1: pcDNA3.1‐ORF10‐flag plasmid
Pep‐DLX6‐AS1‐1: pcDNA3.1‐ORF8‐flag plasmid
Pep‐DLX6‐AS1‐2: pcDNA3.1‐ORF11‐flag plasmid
Pep‐DLX6‐AS1‐3: pcDNA3.1‐ORF12‐flag plasmid
Pep‐KDM4A‐AS1: pcDNA3.1‐ORF3‐flag plasmid
Pep‐KMT2E‐AS1: pcDNA3.1‐ORF5‐flag plasmid
Pep‐KTN1‐AS1: pcDNA3.1‐ORF6‐flag plasmid
Pep‐LINC00665: pcDNA3.1‐ORF4‐flag plasmid
Pep‐LINC00839: pcDNA3.1‐ORF7‐flag plasmid
Pep‐LINC01116: pcDNA3.1‐ORF1‐flag plasmid
Pep‐LMO7‐AS1: pcDNA3.1‐ORF2‐flag plasmid
Pep‐UBL7‐AS1: pcDNA3.1‐ORF9‐flag plasmid
RNA‐seq: RNA‐sequencing
ROC: receiver‐operating characteristic
ROS: reactive oxygen species
SCD: stearoyl‐CoA desaturase
shRNAs: short hairpin RNAs
TOM: topological overlap measure
TRAP: translating ribosome affinity purification
UBL7‐AS1: UBL7 divergent transcript
WGCNA: weighted gene co‐expression network analysis

## 1 Introduction

Esophageal squamous cell carcinoma (ESCC) is the most predominant subtype of esophageal cancer (EC) [1]. Patients with EC have a poor prognosis in the Chinese population, with a 5‐year relative survival of < 40% [2]. Current treatment strategies for EC include surgery, chemotherapy, radiotherapy, molecular targeted therapy, and their combinations [3]. However, ESCC is often diagnosed at the advanced stage and the prognosis of ESCC remains poor [3, 4].

Long noncoding RNAs (lncRNAs), a group of RNAs longer than 200 nucleotides, have been identified as crucial players in the malignant progression of multiple cancers including ESCC [5, 6, 7]. LncRNAs can be categorized into 5 types based on their location relative to protein‐coding genes: antisense RNAs, lincRNAs, sense overlapping transcripts, sense intronic transcripts, and processed transcripts [8]. Moreover, most lncRNAs present tissue‐specific expression [8]. For instance, LINC00680 was highly expressed in ESCC and LINC00680 knockdown hindered ESCC tumorigenesis and progression *in vitro* and *in vivo* [9]. LncRNA lysyl oxidase homolog 1 antisense RNA 1 (LOXL1‐AS1) expression was notably increased in ESCC tissues relative to adjacent normal tissues, and LOXL1‐AS1 depletion markedly weakened the proliferative, migratory, and invasive abilities of ESCC cells and hindered ESCC cell cycle progression [10].

Over the past decades, lncRNAs have been initially described as transcripts that cannot encode proteins or peptides [11, 12]. With the development of bioinformatics and high‐throughput transcriptomics and proteomics approaches including ribosome profiling, ribosome sequencing, and mass spectrometry (MS) analysis, accumulating lncRNAs have been recognized to be RNA molecules with open reading frames (ORFs) and peptide/protein‐coding potential [12, 13, 14]. Moreover, previous studies have demonstrated that some lncRNAs can encode functional peptides to regulate various pathophysiologic processes including carcinoma progression [12, 13, 15]. For instance, lncRNA linc00467 could encode an uncharacterized 94‐amino acid (94‐aa) ATP synthase–associated peptide (ASAP), which facilitated colorectal carcinoma cell proliferation *in vitro* and colorectal carcinoma xenograft tumor growth *in vivo* [16]. LncRNA linc00278 could encode a 21‐aa short peptide and the peptide encoded by linc00278 hindered the growth of ESCC xenograft tumors [17].

In this study, lncRNAs with potential peptide‐coding abilities in ESCC were identified by weighted gene co‐expression network analysis (WGCNA), prior ribosome sequencing analyses, ORF prediction, MS dataset identification, and western blotting validation. Moreover, the effects of peptides encoded by *KDM4A‐AS1* on ESCC cell viability and migration were examined. Additionally, the molecular basis of the *KDM4A‐AS1*‐encoded peptide was preliminarily explored.

## 2 Materials and methods

### 2.1 Plasmid construction

To produce the flag fusion protein constructs of matching lncRNA ORFs in Table S1, the sequences of 12 ORFs were synthesized and constructed into pcDNA3.1 vector with 3 × flag tags at the carbon end to generate Pep‐LINC01116, Pep‐LMO7‐AS1, Pep‐KDM4A‐AS1, Pep‐LINC00665, Pep‐KMT2E‐AS1, Pep‐KTN1‐AS1, Pep‐LINC00839, Pep‐DLX6‐AS1‐1, Pep‐UBL7‐AS1, Pep‐DDX11‐AS1, Pep‐DLX6‐AS1‐2, and Pep‐DLX6‐AS1‐3 recombinant plasmids, respectively. The *KDM4A‐AS1* ORF start codon ‘ATG’ in pcDNA3.1‐ORF3‐flag (Pep‐KDM4A‐AS1) plasmid was mutated to the codon ‘ATT’ to generate the Pep‐KDM4A‐AS1‐mut plasmid. The *KDM4A‐AS1* ORF was also constructed into pcDNA3.1 vector with a GFP tag at the carbon end and the recombinant plasmid was named Pep‐KDM4A‐AS1‐GFP. The start codon ‘ATG’ in Pep‐KDM4A‐AS1‐GFP was mutated to the codon ‘ATT’ to produce the Pep‐KDM4A‐AS1‐GFP‐mut plasmid. Short hairpin RNAs (shRNAs) targeting *KDM4A‐AS1* were constructed into the pLKO.1‐CMC‐copGFP‐puro vector (Qingke, Beijing, China). The target sequences of *KDM4A‐AS1* shRNAs were 5′‐GGATATCAGAGAATAATAA‐3′ and 5′‐AGCAGATTCCACACTGCAACT‐3′.

### 2.2 Cell culture and transfection

KYSE150 (RRID: CVCL_1348), TE‐1 (RRID: CVCL_1759), and human embryonic kidney cell line HEK293T (RRID: CVCL_0063) cells were purchased from Procell Life Science Technology Co., Ltd. (Wuhan, China). KYSE150 and TE‐1 cells were cultured in RPMI‐1640 medium (cat. no. PM150110; Procell) containing 10% fetal bovine serum (FBS; cat. no. 164210‐50; Procell) and 1% penicillin/streptomycin (cat. no. PB180120; Procell). HEK293T cells were grown in DMEM medium (cat. no. PM150210; Procell) supplemented with 10% FBS (cat. no. 164210‐50; Procell) and 1% penicillin/streptomycin (cat. no. PB180120; Procell). All cells were maintained in a 5% CO_2_ incubator at 37 °C. Cell lines were authenticated by short tandem repeat (STR) profiling. STR genotyping was performed as previously described [18, 19]. Briefly, genomic DNA was extracted from KYSE150, TE‐1, and HEK293T cells using the PureLink Genomic DNA Mini kit (Thermo Scientific, Waltham, MA, USA) following the instructions of the manufacturer. Next, multiple PCR amplification was performed using the PowerPlex 21 system (Promega, Madison, WI, USA), and PCR products were detected using the ABI 3730XL Genetic Analyzer (Thermo Scientific). Data were analyzed using the genemapper software (Thermo Scientific). The STR profiles of HEK293T, TE‐1 and KYSE150 cell lines were shown in Table 1. Mycoplasma test experiments were performed using the Mycoplasma PCR Detection Kit (Beyotime Biotechnology, Shanghai, China) according to the protocols of the manufacturer. Mycoplasma testing experiments validated that KYSE150, TE‐1, and HEK293T cells were not contaminated by mycoplasma.

**Table 1:** STR profiles of HEK293T, TE‐1, and KYSE150 cell lines.

Markers	HEK293T STR profile	TE‐1 STR profile	KYSE150 STR profile
Amelogenin	X	X	X
CSF1PO	11,12	10,12	12,13
D2S1338	19	19	25
D3S1358	15,16,17	16	15,16
D5S818	8,9	11	12,13
D7S820	11	10,11	10,11
D8S1179	11,12,14	11,13	10,15
D13S317	12,14	10	8,11
D16S539	9,13	12	9,11
D18S51	17,18	17	14
D19S433	17,18	14,15.2	15,15.2
D21S11	28,30.2	28	30,31
FGA	23	24	21,24
PentaD	9,10	10	10
PentaE	7,15	12,18	12,18
TH01	7,9.3	7	7,9
TPOX	11	8,11	8
vWA	16,19	18,19	16,17
D6S1043	11	11,12	18,20
D12S391	19,21	20	19,22
D2S441	11,15	10,11	10,11

Plasmids were transfected into cells using the Lipofectamine 3000 reagent (Thermo Scientific) following the instructions of the manufacturer. Cells (1 × 10^6^ cells per well) were plated into 6‐well plates and cultured overnight. In the transfection experiments, the Lipofectamine 3000 reagent (5 μL per well) was diluted in 125 μL opti‐MEM medium. Plasmid (2.5 μg per well) was diluted in 125 μL opti‐MEM medium and then mixed with P3000 reagent (5 μL per well). Next, the diluted DNA was added to the diluted Lipofectamine 3000 reagent. After 15 min of incubation at room temperature, the DNA‐lipid complex was added to cells. At the indicated time points after transfection, transfected cells were analyzed.

### 2.3 Data collection, data processing, and differential expression analysis

ESCC RNA‐sequencing (RNA‐seq) dataset containing the information on counts and clinical parameters was downloaded from the TCGA database on the UCSC Xena platform (https://xenabrowser.net/datapages/) [20]. Next, the data of ESCC samples were screened out from the TCGA ESCC RNA‐seq dataset based on the clinical information. The paraffin‐embedded samples (with ‘‐01B’ or ‘‐11B’ suffix) were removed as previously described [21]. Samples with the eleventh, twelfth, and thirteenth letters being 01A or 11A (01: carcinoma samples; 11: normal samples; A: favorable‐quality samples) in the submitter IDs were screened out for further analyses. Accordingly, a total of 88 samples including 77 ESCC samples and 11 normal samples were filtered out. Next, differential expression patterns of genes and lncRNAs in 77 ESCC samples versus 11 normal samples were examined by the deseq2 package with a *P*‐value < 0.05 as statistically significant [22].

### 2.4 WGCNA

Before WGCNA analysis, genes with no expression in over 50% of ESCC samples were removed. WGCNA analysis was carried out using the WGCNA R package as described previously [23, 24]. Briefly, a sample hierarchical clustering analysis was performed to detect outliers. A similarity matrix was constructed based on Pearson correlation coefficients of genes and then turned into an adjacent matrix. Next, the adjacent matrix was converted into a topological matrix at the soft threshold power using the topological overlap measure (TOM). Genes were clustered into different modules through the dynamic tree‐cut algorithm according to the TOM‐based dissimilarity (1‐TOM). Finally, the correlations of modules and ESCC clinical characteristics were analyzed by Pearson correlation coefficient and visualized by heatmap. The correlations were defined to be statistically significant at a *P*‐value < 0.05. Genes in the gray module were considered to be nonspecific genes and were discarded in the subsequent analysis.

### 2.5 Prediction of lncRNAs with peptide‐coding potential

Ribosome footprint datasets SRR1758391 and SRR8550319 were downloaded from the TranslatomeDB database. The longest transcripts of lncRNAs were retrieved from the Ensembl (human GRCh38.p13) website (http://asia.ensembl.org/Homo_sapiens/Gene/Summary?db=core;g=ENSG00000236200;r=1:43685123‐43708138). The ORFs of lncRNAs were predicted by the EMBOSS: getorf website (http://emboss.bioinformatics.nl/cgi‐bin/emboss/getorf) with the parameters as follows: ‘minimum nucleotide size of ORF to report’‐‘15’, ‘type of output’‐‘translation of regions between STRAT and STOP codons’, and ‘find ORFs in the reverse sequence?’‐‘no’. The putative ORF‐encoded peptide sequences were compared against the ESCC MS dataset IPX0002962000 using the MaxQuant software. The MS searching parameters against the ESCC MS dataset IPX0002962000 were displayed in Table S2. Next, the UniProt database was used to exclude the peptide fragments that derived from cleavage products of known human proteins from peptides identified by MS.

### 2.6 Western blotting assay

The recombinant plasmids were transfected into TE‐1 or KYSE150 cells. At 48 h after transfection, cells were collected and lysed using the RIPA lysis buffer (cat. no. P0013B, Beyotime Biotechnology) containing a protease inhibitor cocktail (cat. no. P1010, Beyotime Biotechnology). Protein was quantified using the BCA Protein Assay Kit (cat. no. P0012S, Beyotime Biotechnology) following the manufacturer's protocols. An equal amount of protein samples (40 μg per lane) was loaded onto 15% sodium dodecyl sulfate‐polyacrylamide gel electrophoresis gel and then transferred onto polyvinylidene fluoride membranes (cat. no. G6045‐0.22, Servicebio, Wuhan, China). Next, the membranes were sequentially incubated with blocking buffer (1 h, room temperature), primary antibody against flag (overnight, 4 °C) (1:5000, cat. no. 3064, DAIAN Biotechnology Co., Ltd, Wuhan, China), and horseradish peroxidase‐secondary antibody conjugate (1 h, room temperature) (1:10 000, cat. no. Q1002, DAIAN Biotechnology Co., Ltd). Finally, protein signals were developed using the Ultra‐Sensitive ECL Chemiluminescence Kit (cat. no. G2020‐25mL, Servicebio).

### 2.7 Cell counting Kit‐8 (CCK‐8) assay

Cell viability was measured using the Cell Counting Kit‐8 (CCK‐8) kit (cat. no. C0037, Beyotime Biotechnology) referring to the manufacturer's protocols. Briefly, cells (1 × 10^4^ cells per well) were plated into 96‐well culture microplates and transfected with corresponding plasmids. At 48 h post‐transfection, 10 μL of CCK‐8 solution was added to each well. The microplates containing CCK‐8 solution were incubated for 1 h in a 37 °C incubator. Next, the optical density values were measured at 450 nm.

### 2.8 Wound healing assay

Cells were seeded into 6‐well plates and transfected with corresponding plasmids. When cells reached 100% confluence, straight wounds were scraped on the cell monolayer. After scratching, detached cells were washed, and then the fresh serum‐free medium was replenished into the wells. Wound areas were imaged at 0 and 48 h after scratching. Cell migration areas were analyzed using the imagej software (National Institutes of Health, Bethesda, MD, USA).

### 2.9 Transwell migration assay

Transfected cells (5 × 10^4^ cells per well) were re‐suspended in serum‐free medium and added to the upper compartment of the 8‐μm pores transwell plate (Costar Corning Inc., Corning, NY, USA). Medium supplemented with 10% FBS was added to the lower compartment. After 48 h of incubation, cells on the upper side of the membranes were wiped. Cells on the lower side of the insert membranes were fixed with 4% paraformaldehyde, followed by staining with 0.1% crystal violet solution (Servicebio). Next, migrated cells were photographed under a microscope and counted. Migratory cell number was analyzed using the Image J software (National Institutes of Health).

### 2.10 Establishment of ESCC cells stably transduced with lentiviral particles

The sequence of RPL10A (NM_007104.5) was constructed into a pcSLenti‐CMV‐EGFP‐3xFLAG‐PGK‐puro‐WPRE3 vector and the recombinant plasmid was named lenti‐RPL10A plasmid. Lenti‐RPL10A or empty vector plasmid was transduced into HEK293T cells together with the psPAX2 and pMD2.G plasmids. At 48 and 72 h after transduction, cell supernatants containing lentiviral particles were collected. Lentiviral titer was determined using the following formula: TU/mL = (*C* × *N* × *D* × 1000)/*V*. TU/mL: transducing units per mL. *C*: average viral copy number per genome. *N*: cell number at infection. *D*: lentivirus dilution ratio. *V*: added lentivirus volume (μL). TE‐1 cells were infected with lenti‐RPL10A lentiviral particles. At 48 h after transduction, cells were cultured in the medium containing puromycin. Next, cells stably transduced with lenti‐RPL10A lentiviral particles were established after 1 week of puromycin selection.

### 2.11 Translating ribosome affinity purification (TRAP)

TRAP assay was carried out using the experimental procedures as previously described [25]. Briefly, TE‐1 cells stably transduced with lenti‐RPL10A lentiviral particles were treated with cycloheximide (10 μg·mL^−1^) for 5 min and then lysed. Next, cell lysate supernatants were incubated with protein A/G magnetic beads (cat. no. M0134, DAIAN Biotechnology Co., Ltd), IgG antibody (cat. no. Q6004, DAIAN Biotechnology Co., Ltd) or GFP antibody (cat. no. 2057, DAIAN Biotechnology Co., Ltd), and RNase I (100 U·μL^−1^) for 1 h at room temperature. Beads were incubated with lysis buffer containing 1% SDS and proteinase K for 1 h at 45 °C. Next, RNA was extracted using RNAeasy Animal RNA isolation Kit (cat. no. R0024, Beyotime Biotechnology). The level of *KDM4A‐AS1* was measured by RT‐qPCR assay.

### 2.12 Bioinformatics analysis

TransLnc database (http://bio‐bigdata.hrbmu.edu.cn/TransLnc/) was used to further support the conclusion that *KDM4A‐AS1* could encode Pep‐KDM4A‐AS1 peptide. Hydrophilic‐hydrophobic analysis of *KDM4A‐AS1*‐encoded peptide was performed using the ProtScale database.

### 2.13 Co‐immunoprecipitation (Co‐IP) assay

The Co‐IP assay was conducted using the Anti‐FLAG (DYKDDDDK) FAST IP kit (cat. no. KIP0064, DAIAN Biotechnology) according to the manufacturer's protocols. Briefly, HEK293T cells were transfected with Pep‐KDM4A‐AS1. At 48 h after transfection, cell lysates were prepared using the ice‐cold lysis buffer containing protease inhibitors. After ultrasonication and centrifugation, cell supernatants were collected. A small volume of lysates was placed into a new centrifuge tube as the input samples. The remaining lysates were divided into two equal parts and incubated overnight at 4 °C with anti‐IgG or anti‐flag affinity gel, respectively. Next, the loading buffer was added to the above gel samples and input samples. The mixtures were boiled for 5 min at 100 °C. After centrifugation, the supernatants were loaded into the wells of 15% SDS/PAGE gel to separate the protein. Then, protein in the SDS/PAGE gel was developed using the Fast Silver Stain Kit (cat. no. P0017S, Beyotime Biotechnology) following the manufacturer's instructions.

### 2.14 MS analysis

Prior to MS analysis, protein lysates were added with DTT (final concentration: 5 mm) for 1 h at 37 °C. When the temperature declined to room temperature, 10 mm iodoacetamide was added to the above system and incubated for 45 min at room temperature under dark conditions. Next, samples were diluted fourfold with 25 mm ammonium bicarbonate and digested overnight with trypsin at the ratio of 1:50 (trypsin:protein) at 37 °C. On the next day, formic acid was added until the pH was < 3. Next, samples were desalted using C18 columns, which were activated by 100% acetonitrile and equilibrated using 0.1% formic acid before loading samples. Then, the columns were washed using 0.1% formic acid, and peptide fragments were eluted using 70% acetonitrile and lyophilized. Next, the lyophilized powder was dissolved in 10 μL of solution A (100% water, 0.1% formic acid) and then centrifuged at 14 000 ***g*** for 20 min at 4 °C. Subsequently, supernatants were collected and injected into the RIGOL L‐3000 High‐Performance Liquid Chromatography system (RIGOL TECHNOLOGIES, INC., Beijing, China). Next, gradient elution was carried out at a flow rate of 600 nL·min^−1^. The elution condition was shown in Table 2. Subsequently, MS data were captured using the Orbitrap Exploris 480 mass spectrometer (Thermo Scientific) with FAIMS Pro Interface, Nanospray Flex (NSI) ion source, and switched compensation voltage CV per second at −45 and −65 V in the data‐dependent acquisition (DDA) mode.

**Table 2:** Elution gradients of liquid chromatography.

Time (min)	Percentage of mobile phase solution B (80% acetonitrile, 0.1% formic acid)
0	6
5	12
38	30
45	40
46	95
60	95

MS scanning range was set to *m*/*z* 350–1200. The first‐order mass spectra (MS1) were collected with a resolution of 60 000 (200 *m*/*z*), custom AGC, and C‐trap maximum injection time of 50 ms. The second mass spectra (MS2) were scanned with a resolution of 15 000 (200 *m*/*z*), custom AGC, maximum injection time of 22 ms, and peptide fragmentation collision energy of 30% in the ‘Top Speed’ mode. Next, the raw MS/MS file data were searched against the homo sapiens database using the proteome discoverer v2.4 software (Thermo Scientific). The search parameters were shown in Table 3. To further remove nonspecific proteins among proteins after Co‐IP experiments and MS analysis, we used the CRAPome database (https://reprint‐apms.org/?q=chooseworkflow) to discard the potentially contaminated proteins in the flag tag AP‐MS. This experimental strategy was also used in a previous study [26].

**Table 3:** Proteome Discoverer 2.4 searching parameters.

Parameters	Value
Enzyme	Trypsin
Static modification	Carbamidomethyl(C)
Dynamic modification	M Oxidation (15.995 Da); Acetyl (Protein N terminus)
Precursor ion mass tolerance	± 15 ppm
Fragment ion mass tolerance	± 0.02 Da
Max missed cleavages	2

### 2.15 GO and KEGG enrichment analysis

GO and KEGG enrichment analysis was performed using the r software (version 3.6.3, The R Foundation for Statistical Computing, Vienna, Austria), clusterprofiler r package (version 3.14.3), and org.hs.eg.db package (version 3.10.0). The Circos plot was drawn to visualize gene associations with the representative GO molecular function terms or KEGG pathways as previously described [27, 28].

### 2.16 Receiver operating characteristic (ROC) analysis

TCGA esophageal cancer RNA‐seq data containing the clinical information were downloaded from the GDC Data Portal platform [29]. The diagnostic values of 10 lncRNAs in EC were evaluated by the area under the curve (AUC) of the ROC curve using the software r (Version 3.6.3) and proc (Version 1.17.0.1, The R Foundation for Statistical Computing).

### 2.17 RT‐qPCR assay

Total RNA extraction was conducted using TRI Reagent Solution (cat. no. AM9738, Thermo Scientific), and the complementary DNA (cDNA) first strand was synthesized by RevertAid First Strand cDNA Synthesis Kit (cat. no. K1621, Thermo Scientific) based on the instructions of the manufacturer. Next, quantitative analysis was performed using the SYBR Select Master Mix (cat. no. 4472919, Thermo Scientific) and corresponding specific primers. The primer sequences were presented as follows: *KDM4A‐AS1* (Forwards: TTGGACCCTGGAAGCACCTA; Reverse: CTTTCCTTTGTGTGCAGGTGG); SCD (Forwards: GGAGCCACCGCTCTTACAAA; Reverse: GAAAACTTGTGGTGGGCACG); ACOX1 (Forwards: CGCCGAGAGATCGAGAACAT; Reverse: CTGTCTGGGCATAAGTGCCA); FASN (Forwards: AGGAACTCCCCTCATCTCCC; Reverse: TTGCCGTTCTCTGACACCTC). β‐actin was used to normalize the expression of other genes. Relative expression levels of genes were calculated using the $2^{-\Delta \Delta C_{t}}$ method.

### 2.18 Immunofluorescence (IF) assay

KYSE150 and TE‐1 cells were transfected with pcDNA3.1 or Pep‐KDM4A‐AS1. At 48 h after transfection, cells climbing slides were permeabilized with permeabilization solution (Servicebio) and blocked with 3% BSA. Next, cells were incubated with anti‐FASN primary antibody (cat. no. 10624‐2‐AP, Proteintech, Wuhan, China) for almost 12 h at 4 °C and FITC‐conjugated goat‐anti‐mouse secondary antibody (cat.no. GB22301, Servicebio) for 1 h at room temperature. Cell nuclei were stained with DAPI (Servicebio) for 10 min at room temperature. Finally, cells were imaged through fluorescent microscopy.

### 2.19 Reactive oxygen species (ROS) detection

KYSE150 and TE‐1 cells were transfected with pcDNA3.1 or Pep‐KDM4A‐AS1. At 48 h after transfection, the ROS level was measured using the ROS Assay Kit (cat.no. S0033S, Beyotime Biotechnology) following the protocols of the manufacturer.

### 2.20 Mitochondrial membrane potential (MMP) detection

KYSE150 and TE‐1 cells were transfected with pcDNA3.1 or Pep‐KDM4A‐AS1. At 48 h after transfection, MMP was examined using the Mitochondrial membrane potential assay kit with JC‐1 (cat.no. C2006, Beyotime Biotechnology) according to the instructions of the manufacturer.

### 2.21 Statistical analysis

Unless otherwise indicated, mean ± SD from at least 3 independent replicates was shown in the graphs. Statistical analysis was carried out by graphpad prism software (Version 7; La Jolla, CA, USA). Difference comparisons were conducted by the Student's *t*‐test (for two‐group data) or one‐way analysis of variance (ANOVA) together with the Tukey test. The statistical significance was defined at a *P*‐value < 0.05.

## 3 Results

### 3.1 Identification of genes and lncRNAs related to histologic grades and TNM stages by WGCNA in ESCC

**Fig. 1:**
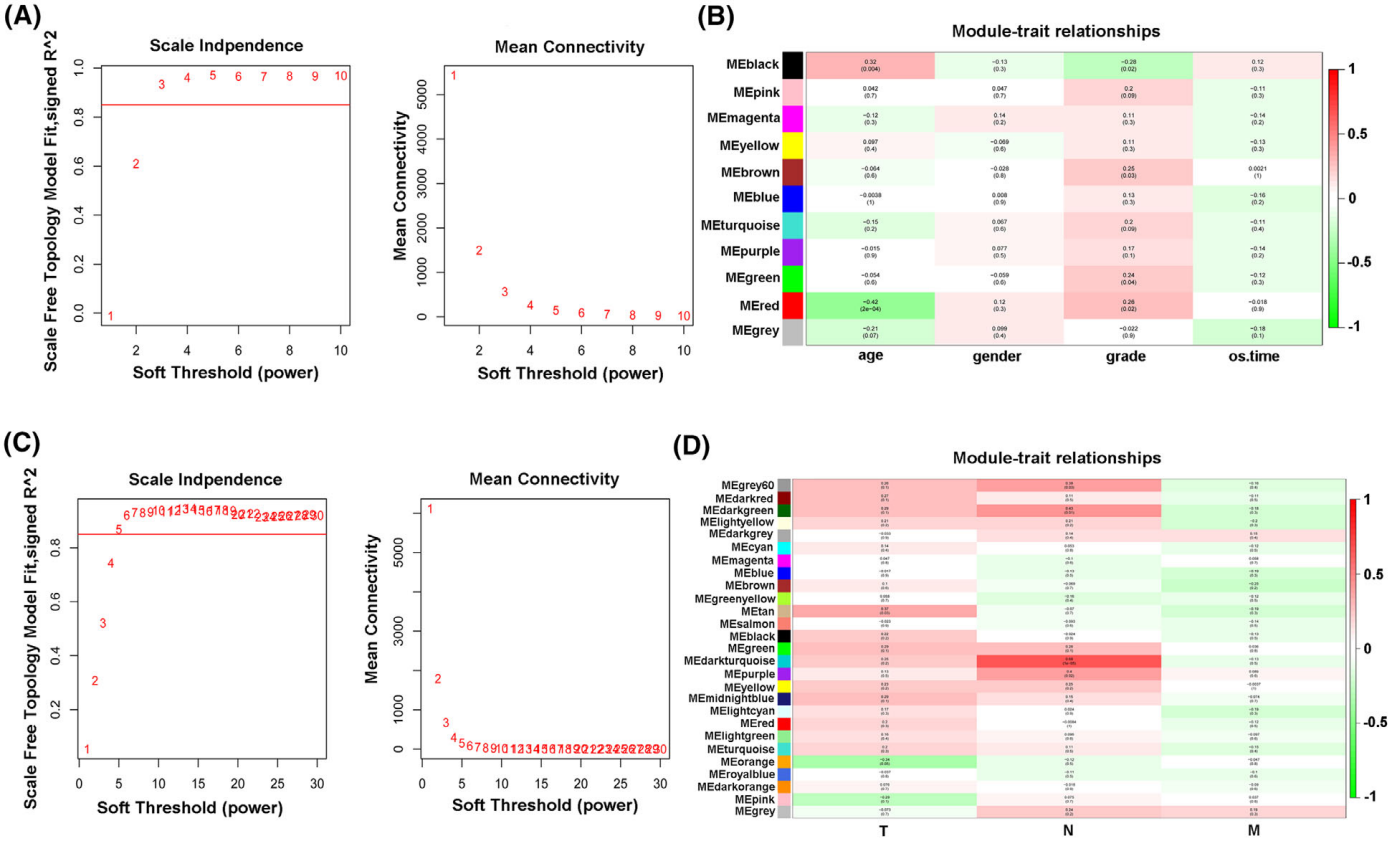
Identification of genes and lncRNAs related to histologic grades and TNM stages by WGCNA in ESCC. (A) Identification of the soft threshold power value to construct the WGCNA network of modules and age/gender/tumor pathological grade/overall survival time of 75 ESCC patients. The soft threshold was set at 3 because of the trade‐off between maximizing scale‐free topology model fit (*R* ^2^) and maintaining high connectivity. The red horizontal line indicates scale‐free topology fit index *R* ^2^ = 0.85. The red Arabic numerals represent different soft threshold values. (B) The correlation heatmap of module eigengene values and age/gender/tumor pathological grade/overall survival time of 75 ESCC patients. (C) Identification of the soft threshold power value in the WGCNA analysis of modules and Tumor (T)/Node (N)/Metastasis (M) stages of 33 ESCC patients. The soft threshold was set at 5 due to the trade‐off between maximizing *R* ^2^ and maintaining high connectivity. The red horizontal line indicates scale‐free topology fit index *R* ^2^ = 0.85. The red Arabic numerals represent different soft threshold values. (D) Correlation matrix for module eigengene values and pathologic Tumor (T)/Node (N)/Metastasis (M) stages. ESCC—esophageal squamous cell carcinoma; LncRNA—long noncoding RNA; TNM stages—tumor (T)/node (N)/metastasis (M) stages; WGCNA—weighted gene co‐expression network analysis.

Among the above‐mentioned 77 TCGA ESCC samples, 75 ESCC samples containing clinical information on histological grades and overall survival time were filtered out for subsequent WGCNA analysis. As shown in Fig. 1A, the soft threshold power was set to 3 (scale‐free topology model fit *R* ^2^ > 0.85) to ensure a scale‐free network. After merging the similar modules at the clustering height cutoff of 0.25, 10 modules were identified (Fig. 1B). Correlation analysis of modules and clinical traits revealed that the black (*R* = −0.28, *P*‐value = 0.02), brown (*R* = 0.25, *P*‐value = 0.03), green (*R* = 0.24, *P*‐value = 0.04), and red (*R* = 0.26, *P*‐value = 0.02) modules were correlated with the histologic grades of ESCC (Fig. 1B). Moreover, WGCNA analysis for 33 TCGA ESCC samples with the complete information on T, N, and M stages was conducted at the soft threshold power of 5 (scale‐free topology model fit *R* ^2^ > 0.85) and the clustering height cutoff of 0.2 (Fig. 1C). Results showed that tan module (*R* = 0.37, *P*‐value = 0.03) was correlated with T stage (Fig. 1D). And, the grey60 (*R* = 0.38, *P*‐value = 0.03), darkgreen (*R* = 0.43, *P*‐value =0.01), darkturquoise (*R* = 0.68, *P*‐value = 1e‐5), or purple (*R* = 0.4, *P*‐value = 0.02) modules were associated with N stage (Fig. 1D). Given the close link between histological grades/TNM stages and ESCC malignant progression, genes in the modules related to TNM stages or histological grades were considered to be crucial in ESCC development.

### 3.2 Identification of lncRNAs with potential translation ability in ESCC

Recently, accumulating evidence shows that some lncRNAs have potential protein/peptide‐coding abilities. Thus, lncRNAs with potential translation capacities were examined in our project. Due to the difficulty in the obtainment of the ribosome sequencing and ribosome footprint data in ESCC, ribosome footprint datasets SRR1758391 and SRR8550319 were downloaded from the TranslatomeDB database to identify lncRNAs that could bind with ribosomes. In combination with lncRNAs in SRR1758391 and SRR8550319 datasets and lncRNAs associated with histological grades in the WGCNA analysis, 118 common lncRNAs were identified. Next, 3793 potential ORFs were identified on the longest transcripts of 118 lncRNAs by ORF prediction analysis. The information about these lncRNA ORFs‐matched peptides was shown in Table S3. Next, these putative ORFs‐encoded peptide sequences were compared against the ESCC MS dataset IPX0002962000. Among these ORFs‐encoded peptides, 29 peptides related to ESCC histological grades were identified in the ESCC MS dataset IPX0002962000. The information about these 29 peptides related to ESCC histological grades was presented in Table S4.

Combined with lncRNAs in SRR1758391 and SRR8550319 datasets and lncRNAs related to TNM stages in the WGCNA analysis, 144 common lncRNAs were identified. ORF prediction analysis suggested that there were 4746 potential ORFs on the longest transcripts of the above 144 lncRNAs. The information on these ORFs‐corresponding peptides was displayed in Table S5. Next, nine peptide fragments related to ESCC TNM stages were identified in the ESCC MS dataset IPX0002962000, which was shown in Table S6. To examine whether the peptide segments identified by MS are derived from cleavage products of known human proteins or not, all of the peptide segments searched by MS were imported into the Blast menu of UniProtKB reference proteomes plus Swiss‐Prot database. The blast results showed that no peptide identified by MS was homologous with any known protein in the database, suggesting that these peptides were previously uncharacterized in humans. Among the peptides that were encoded by lncRNAs related to ESCC histological grades and TNM stages, 12 peptides were screened out for further investigations under the conditions: (a) 25‐aa < length of peptide < 100‐aa; (b) *P*‐value in the differential expression analysis < 0.05. The information on these 12 potential peptides and peptides‐matched ORFs/lncRNAs was displayed in Table S1. The screening flowchart of lncRNAs with peptide‐coding potential and associations with ESCC progression was shown in Fig. S1.

### 3.3 Diagnostic values, expression analysis, and encoding ability validation of the above lncRNAs.

**Fig. 2:**
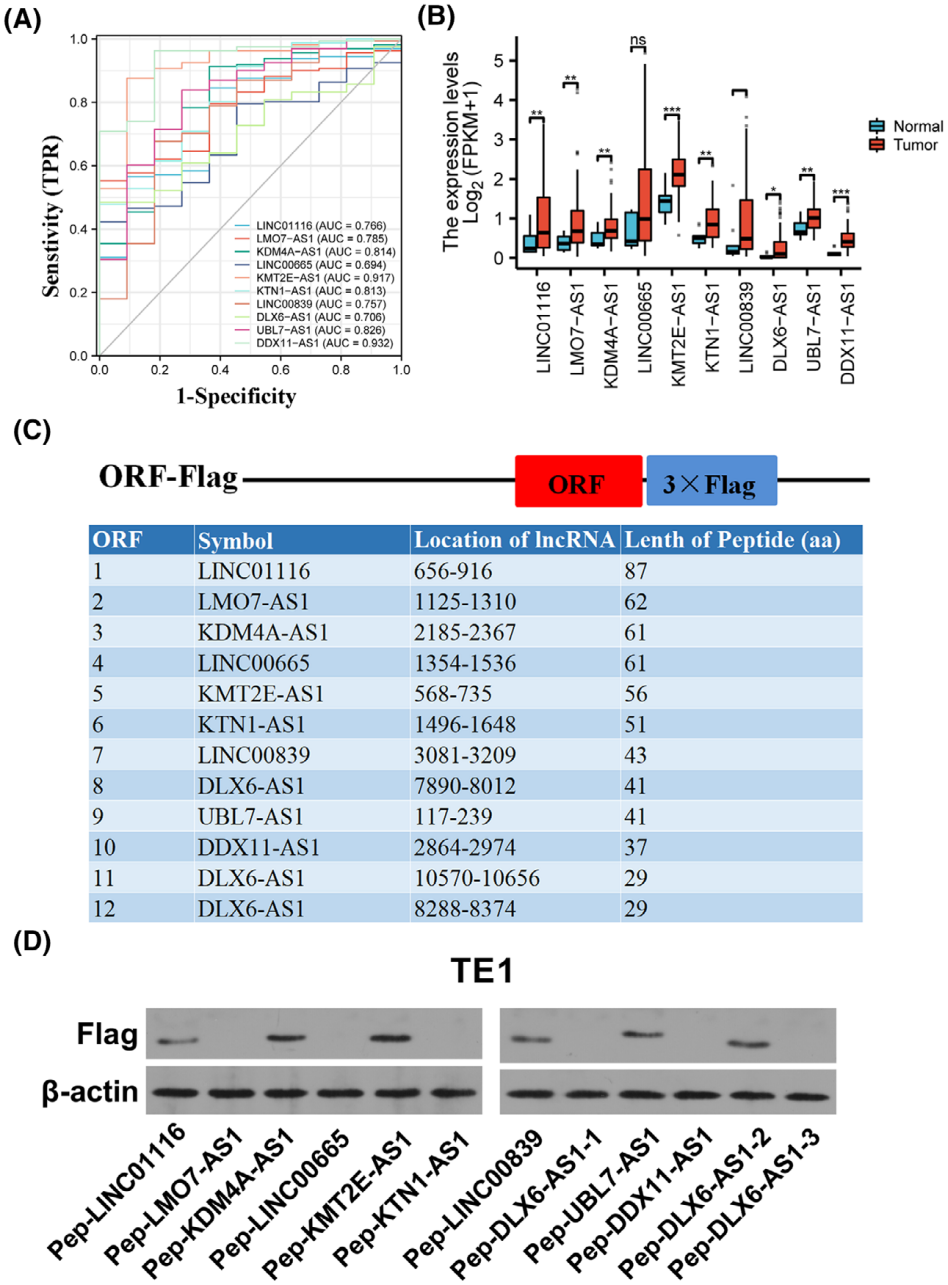
Diagnostic values, expression analysis, and encoding ability validation of the above lncRNAs with peptide‐coding potential and associations with ESCC progression (i.e., *LINC01116*, *LMO7‐AS1*, *KDM4A‐AS1*, *LINC00665*, *KMT2E‐AS1*, *KTN1‐AS1*, *LINC00839*, DLX6‐AS1, UBL7‐AS1, and DDX11‐AS1) in ESCC. (A) ROC curves for *LINC01116*, *LMO7‐AS1*, *KDM4A‐AS1*, *LINC00665*, *KMT2E‐AS1*, *KTN1‐AS1*, *LINC00839*, *DLX6‐AS1*, *UBL7‐AS1*, and *DDX11‐AS1* in ESCC. FPR—false positive rate; TPR—true positive rate. The TCGA EC RNA‐seq data were obtained from the GDC Data Portal platform. (B) The differential expression profiles of *LINC01116*, *LMO7‐AS1*, *KDM4A‐AS1*, *LINC00665*, *KMT2E‐AS1*, *KTN1‐AS1*, *LINC00839*, *DLX6‐AS1*, *UBL7‐AS1*, and *DDX11‐AS1* in 162 TCGA EC samples than in the 11 normal samples. The statistical analysis and data visualization were performed using the software r (Version 3.6.3) and ggplot2 (Version 3.3.3). The data analysis was performed using the Student's *t*‐test and results were shown as mean ± SD. *P < 0.05; **P < 0.01; and ***P < 0.001 vs. normal control group. (C) The diagram of the ORF cloning site and the information on ORF‐matched lncRNA symbols, ORF location on corresponding lncRNAs, and the length of ORF‐translated peptides. (D) TE‐1 cells were transfected with Pep‐LINC01116, Pep‐LMO7‐AS1, Pep‐KDM4A‐AS1, Pep‐LINC00665, Pep‐KMT2E‐AS1, Pep‐KTN1‐AS1, Pep‐LINC00839, Pep‐DLX6‐AS1‐1, Pep‐UBL7‐AS1, Pep‐DDX11‐AS1, Pep‐DLX6‐AS1‐2, and Pep‐DLX6‐AS1‐3. At 48 h after transfection, western blotting assay was performed using the primary antibody against flag tags (*n* = 3). DDX11‐AS1—DDX11 antisense RNA 1; DLX6‐AS1—DLX6 antisense RNA 1; EC—esophageal cancer; ESCC—esophageal squamous cell carcinoma; *KDM4A‐AS1*—lysine‐specific demethylase 4A antisense RNA 1; KMT2E‐AS1—KMT2E antisense RNA 1; KTN1‐AS1—KTN1 antisense RNA 1; LINC00665—long intergenic non‐protein‐coding RNA 665; LINC00839—long intergenic non‐protein‐coding RNA 839; LINC01116—long intergenic non‐protein‐coding RNA 1116; LMO7‐AS1—LMO7 antisense RNA 1; LncRNA—long noncoding RNA; ORF—open reading frame; Pep‐DDX11‐AS1—pcDNA3.1‐ORF10‐flag plasmid; Pep‐DLX6‐AS1‐1—pcDNA3.1‐ORF8‐flag plasmid; Pep‐DLX6‐AS1‐2—pcDNA3.1‐ORF11‐flag plasmid; Pep‐DLX6‐AS1‐3—pcDNA3.1‐ORF12‐flag plasmid; Pep‐KDM4A‐AS1—pcDNA3.1‐ORF3‐flag plasmid; Pep‐KMT2E‐AS1—pcDNA3.1‐ORF5‐flag plasmid; Pep‐KTN1‐AS1—pcDNA3.1‐ORF6‐flag plasmid; Pep‐LINC00665—pcDNA3.1‐ORF4‐flag plasmid; Pep‐LINC00839—pcDNA3.1‐ORF7‐flag plasmid; Pep‐LINC01116—pcDNA3.1‐ORF1‐flag plasmid; Pep‐LMO7‐AS1—pcDNA3.1‐ORF2‐flag plasmid; Pep‐UBL7‐AS1—pcDNA3.1‐ORF9‐flag plasmid; ROC—receiver‐operating characteristic; UBL7‐AS1—UBL7 divergent transcript.

Here, we confirmed that the high diagnostic efficacy of *KDM4A‐AS1* (AUC = 0.814), *KMT2E‐AS1* (AUC = 0.917), *KTN1‐AS1* (AUC = 0.813), *UBL7‐AS1* (AUC = 0.826), and *DDX11‐AS1* (AUC = 0.932) had higher AUC values in EC by ROC analysis (Fig. 2A). Moreover, differential expression analysis suggested that the levels of *LINC01116*, *LMO7‐AS1*, *KDM4A‐AS1*, *KMT2E‐AS1*, *KTN1‐AS1*, *LINC00839*, *DLX6‐AS1*, *UBL7‐AS1*, *and DDX11‐AS1* were notably up‐regulated in 77 ESCC samples (Table S1) or 162 EC tumor tissues (Fig. 2B) than in 11 normal tissues. To examine whether these putative lncRNA ORFs could encode short peptides or not, the sequences of these ORFs were subcloned into pcDNA3.1 vector containing triplicate flag tags, respectively. The diagram of the ORF cloning site was shown in Fig. 2C. Also, ORF‐matched lncRNA symbols, ORF location on corresponding lncRNAs, and the length of ORF‐translated peptides were displayed in Fig. 2C. Subsequent western blotting assay using the antibody against flag demonstrated that *LINC01116*, *KDM4A‐AS1*, *KMT2E‐AS1*, *LINC00839*, *UBL7‐AS1*, *and DLX6‐AS1* could translate into small peptides in TE‐1 cells (Fig. 2D).

### 3.4 Effects of LINC01116‐ and KDM4A‐AS1‐encoded peptides on ESCC cell viability and migration.

**Fig. 3:**
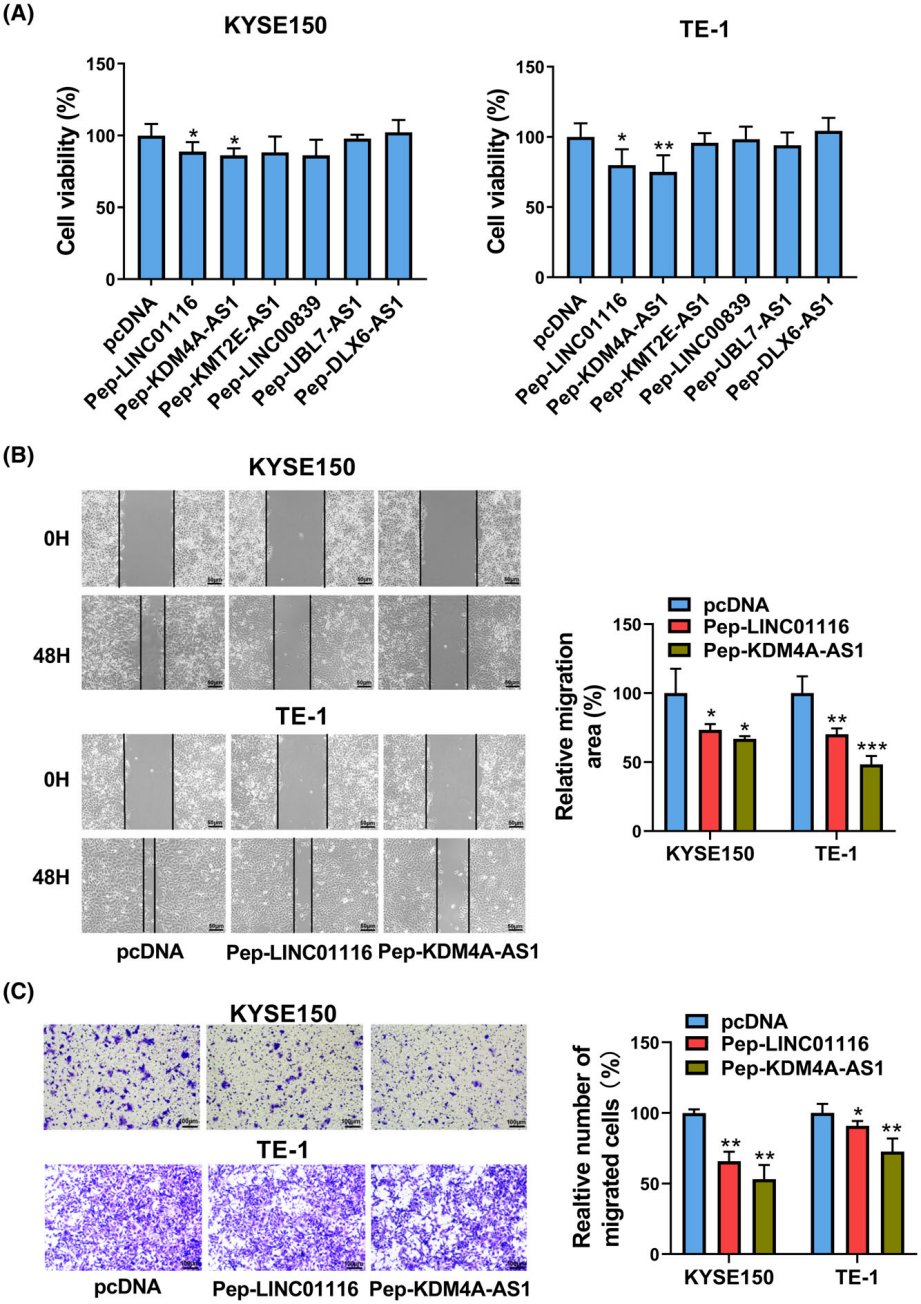
Effects of LINC01116‐ and *KDM4A‐AS1*‐encoded peptides on ESCC cell viability and migration. (A) KYSE150 and TE‐1 cells were transfected with Pep‐LINC01116, Pep‐KDM4A‐AS1, Pep‐KMT2E‐AS1, Pep‐LINC00839, Pep‐UBL7‐AS1, and Pep‐DLX6‐AS1‐2. Then, cell viability was examined by CCK‐8 assay at 48 h post‐transfection (*n* = 5). (B) KYSE150 and TE‐1 cells were transfected with pcDNA3.1, Pep‐LINC01116, or Pep‐KDM4A‐AS1. When cells were grown to approximately 100% confluency, the wounds were created. The wound regions were photographed at 0 and 48 h after scratching (*n* = 3). Scale bar, 50 μm. (C) KYSE150 and TE‐1 cells transfected with pcDNA3.1, Pep‐LINC01116, or Pep‐KDM4A‐AS were seeded into the transwell chambers. At 48 h after transfection, migrated cell number was calculated (*n* = 3). Scale bar, 100 μm. CCK‐8—Cell Counting Kit‐8; ESCC—esophageal squamous cell carcinoma; *KDM4A‐AS1*—lysine‐specific demethylase 4A antisense RNA 1; LINC01116—long intergenic non‐protein‐coding RNA 1116; Pep‐DLX6‐AS1‐2—pcDNA3.1‐ORF11‐flag plasmid; Pep‐KDM4A‐AS1—pcDNA3.1‐ORF3‐flag plasmid; Pep‐KMT2E‐AS1—pcDNA3.1‐ORF5‐flag plasmid; Pep‐LINC00839—pcDNA3.1‐ORF7‐flag plasmid; Pep‐LINC01116—pcDNA3.1‐ORF1‐flag plasmid; Pep‐UBL7‐AS1—pcDNA3.1‐ORF9‐flag plasmid. The data were analyzed by the ANOVA and Tukey test, and the results were presented as mean ± SD from at least three independent repeats. *P < 0.05; **P < 0.01; and ***P < 0.001 vs. pcDNA control group.

CCK‐8 assay showed that the overexpression of peptide that was encoded by LINC01116 (Pep‐LINC01116) led to approximately 12% or 20% reduction in the viability of KYSE150 and TE‐1cells, respectively (Fig. 3A). Also, *KDM4A‐AS1*‐encoded peptide (Pep‐KDM4A‐AS1) triggered 15% or 25% decrease in the viability of KYSE150 and TE‐1cells, respectively (Fig. 3A). The ectopic expression of peptides encoded by *KMT2E‐AS1*, *LINC00839*, *UBL7‐AS1*, *and DLX6‐AS1* did not influence KYSE150 and TE‐1 cell viability (Fig. 3A). The wound healing assay disclosed that the enforced expression of peptide that was encoded by LINC01116 (Pep‐LINC01116) led to an about 35% or 43% reduction in cell migration area in KYSE150 or TE‐1 cells, respectively (Fig. 3B). Cell migration area reduced approximately 53% in both KYSE150 and TE‐1 cells after the overexpression of peptides derived from *KDM4A‐AS1* (Pep‐KDM4A‐AS1; Fig. 3B). Transwell migration assay revealed that Pep‐LINC01116 overexpression triggered an about 30% or 10% reduction in the number of migrated cells in KYSE150 and TE‐1 cells (Fig. 3C). Cell migration number decreased 47% or 27% in KYSE150 and TE‐1 cells following the enforced expression of Pep‐KDM4A‐AS1, respectively (Fig. 3C). These data suggested that peptide that was encoded by *LINC01116* or *KDM4A‐AS1*, especially *KDM4A‐AS1*‐encoded peptide, could reduce ESCC cell viability and inhibit ESCC cell migration.

### 3.5 Further validation of KDM4A‐AS1 coding ability and hydrophilic‐hydrophobic analysis of KDM4A‐AS1‐coded peptide

**Fig. 4:**
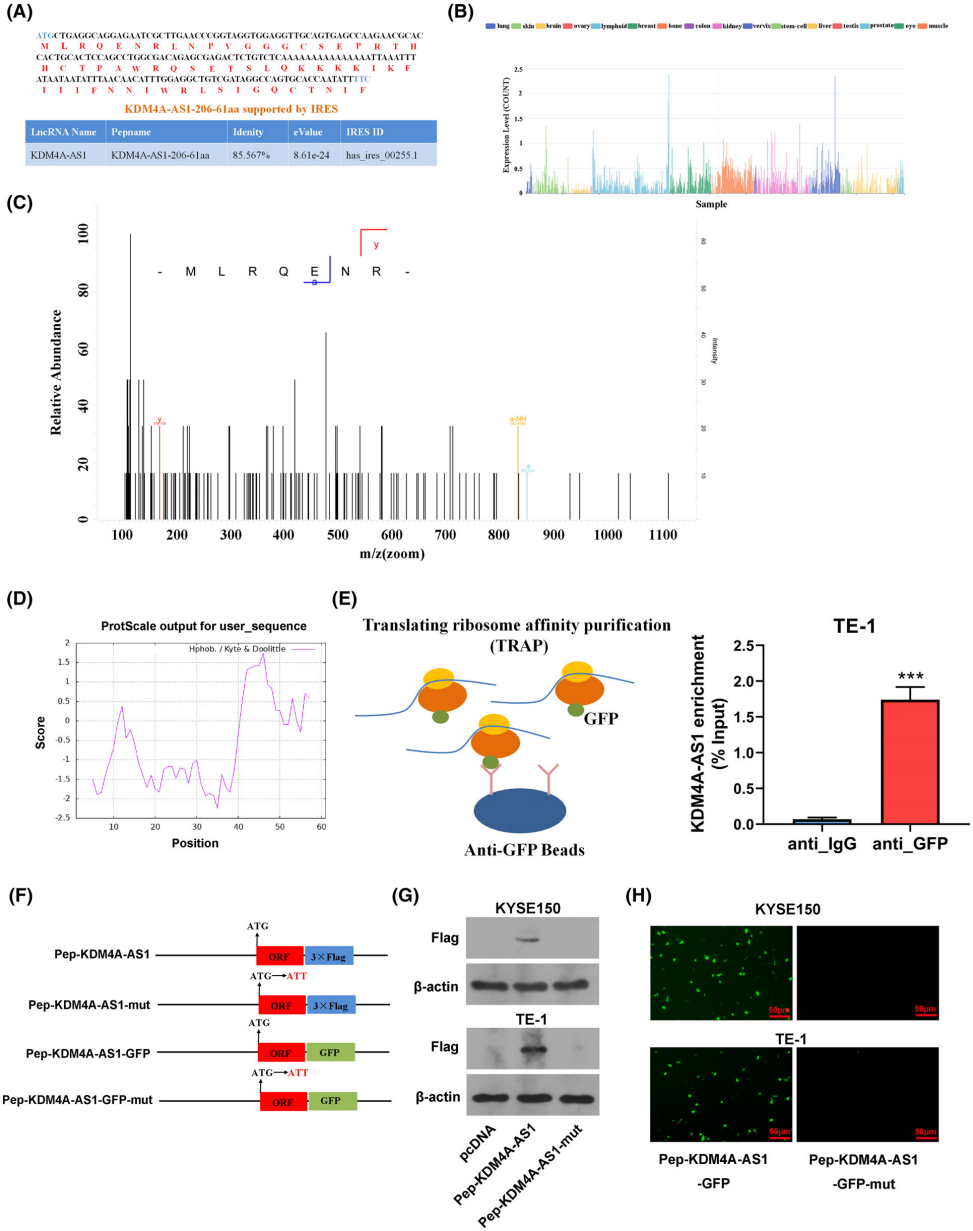
Further validation of *KDM4A‐AS1* coding ability and hydrophilic‐hydrophobic analysis of *KDM4A‐AS1*‐coded peptide. (A) The sequences of *KDM4A‐AS1*‐206‐61aa peptide and IRES evidence of *KDM4A‐AS1*‐encoded peptide (*KDM4A‐AS1*‐206‐61aa). (B) The coding ability of *KDM4A‐AS1* was supported by the occupancy of ribosomes. (C) The MS identification outcome of *KDM4A‐AS1*‐encoded peptide. (D) The ProtScale output for *KDM4A‐AS1*‐encoded peptide (*KDM4A‐AS1*‐206‐61aa). The value of score = −0.559 was obtained by the ProtScale website based on the hydrophilic or hydrophobic score of all amino acids. A score < 0 represented that the peptide was hydrophilic [56]. (E) TE‐1 cells were stably transduced with or without lenti‐RPL10A lentiviral particles. TRAP assay was performed using IgG or GFP antibody in TE‐1 cells stably transduced with or without lenti‐RPL10A lentiviral particles. Next, the *KDM4A‐AS1* enrichment level was measured by RT‐qPCR assay. The data analysis was performed using the Student's *t*‐test and results were shown as mean ± SD from 3 independent repeats (*n* = 3). ***P < 0.001 vs. anti_IgG control group. (F) The structure schematics of Pep‐KDM4A‐AS, Pep‐KDM4A‐AS‐mut, Pep‐KDM4A‐AS‐GFP, and Pep‐KDM4A‐AS‐GFP‐mut. (G) KYSE150 and TE‐1 cells were transfected with pcDNA3.1 empty vector, Pep‐KDM4A‐AS1 or Pep‐KDM4A‐AS1‐mut plasmid. At 48 h post‐transfection, the KDM4A‐AS1‐flag fusion protein levels were measured by western blotting using the anti‐flag antibody. (H) KYSE150 and TE‐1 cells were transfected with Pep‐KDM4A‐AS‐GFP or Pep‐KDM4A‐AS‐GFP‐mut. At 48 h after transfection, GFP fluorescence was detected using a fluorescent microscope. Scale bar, 50 μm. IRES—internal ribosome entry site; *KDM4A‐AS1*—lysine‐specific demethylase 4A antisense RNA 1; MS—mass spectrometry; TRAP—translating ribosome affinity purification.

Next, the lncRNA *KDM4A‐AS1*‐translated peptide was screened out for further investigation due to its stronger inhibitory activity on ESCC cell progression compared with the LINC01116‐encoded peptide. Pep‐KDM4A‐AS1 peptide (*i.e.*, KDM4A‐AS1‐206‐61aa) was also identified in the TransLnc database (http://bio‐bigdata.hrbmu.edu.cn/TransLnc/detail_peptide.jsp?peptide=KDM4A‐AS1‐206‐61aa&species=Human). Also, TransLnc database suggested that Pep‐KDM4A‐AS1 peptide‐corresponding RNA sequence contained an internal ribosome entry site (IRES) element (Fig. 4A). Moreover, the coding potential of *KDM4A‐AS1* was further supported by occupancy of ribosomes in the TransLnc database, which disclosed that *KDM4A‐AS1* could bind with ribosomes in multiple tissues (Fig. 4B). The MS identification outcome of *KDM4A‐AS1*‐encoded peptide was also shown in Fig. 4C. Hydrophilic‐hydrophobic analysis by the ProtScale database revealed that *KDM4A‐AS1*‐encoded peptide had a stronger hydrophilia (score = −0.559) (Fig. 4D). TRAP and RT‐qPCR analyses further validated that lncRNA *KDM4A‐AS1* could bind with ribosomal protein RPL10A in TE‐1 cells (Fig. 4E), which further suggested that *KDM4A‐AS1* had translation potential. To further examine the translation ability of putative *KDM4A‐AS1* ORF, the start codon ‘ATG’ of the putative *KDM4A‐AS1* ORF in the pcDNA3.1‐*KDM4A‐AS1* ORF‐flag fusion plasmid (Pep‐KDM4A‐AS1) was mutated to ‘ATT’ (Fig. 4F). Western blotting validated the abundant expression of *KDM4A‐AS1* ORF‐flag fusion protein in KYSE150 and TE‐1 cells transfected with Pep‐KDM4A‐AS1 (Fig. 4G). However, the mutation of the start codon abolished the translation ability of the putative *KDM4A‐AS1* ORF in KYSE150 and TE‐1 cells (Fig. 4G). Also, putative *KDM4A‐AS1* ORF was subcloned into the pcDNA 3.1 vector carrying the GFP reporter gene (Fig. 4F), and the start codon ‘ATG’ of putative *KDM4A‐AS1* ORF was mutated to ‘ATT’ in the ORF‐GFP fusion plasmid (Fig. 4F). Fluorescence analysis showed that there were noticeable fluorescence signals in KYSE150 and TE‐1 cells transfected with wild‐type ORF‐GFP fusion plasmid, while the fluorescence signals were almost undetectable after the mutation of the start codon ‘ATG’ of putative *KDM4A‐AS1* ORF (Fig. 4H).

### 3.6 Identification of KDM4A‐AS1‐encoded peptide‐related proteins and functions/pathways in ESCC

**Fig. 5:**
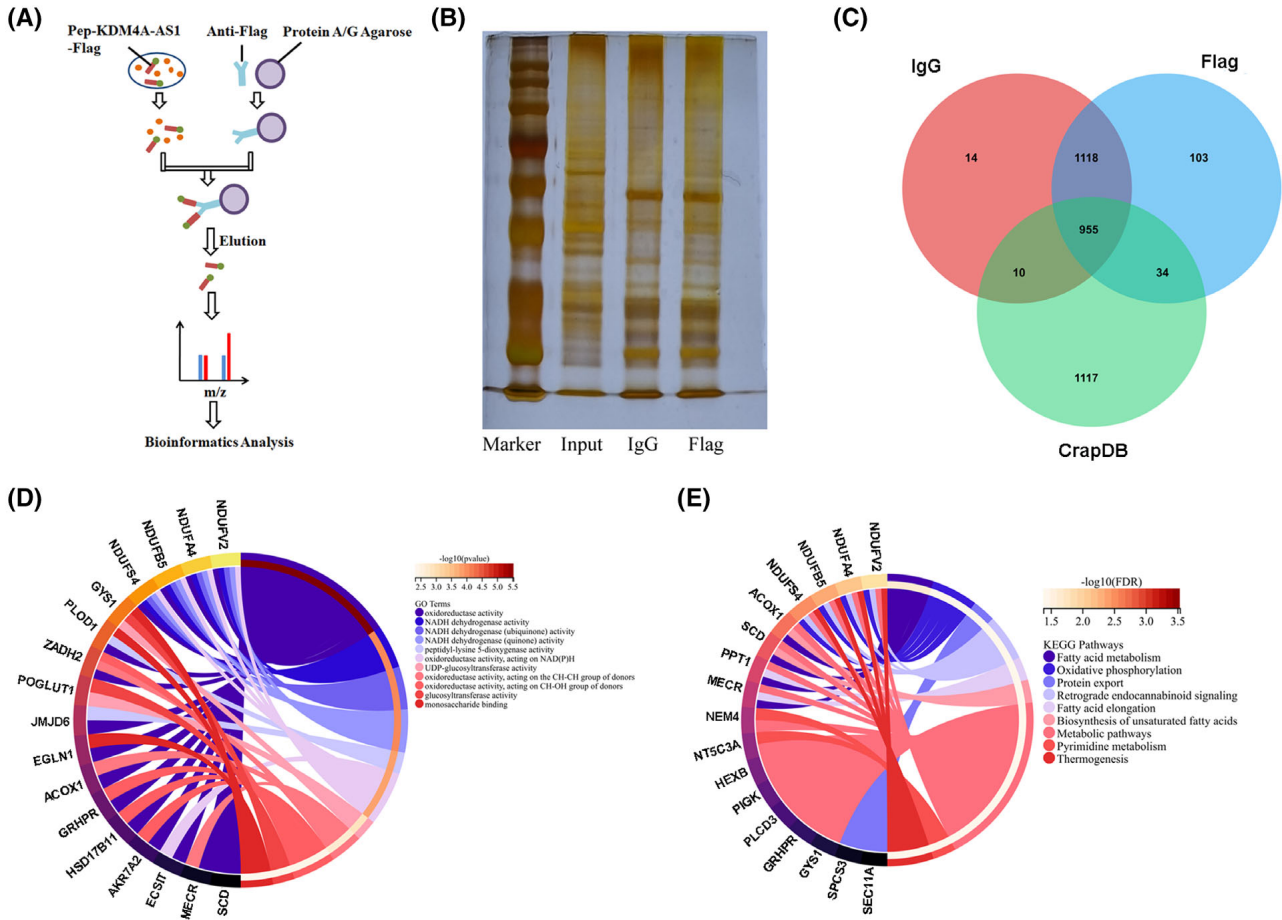
Identification of *KDM4A‐AS*‐encoded peptide‐related proteins and functions/pathways in ESCC. (A) The schematic diagram of Co‐IP. (B) HEK293T cells were transduced with Pep‐KDM4A‐AS1. At 48 h after transduction, the Co‐IP assay was conducted using the IgG or flag antibody (*n* = 1). Next, proteins in the input sample, IgG group, and flag group were analyzed by silver staining. (C) Venn analysis for proteins identified in IgG or flag group after Co‐IP and MS analysis and CRAPome‐identified nonspecific proteins in the flag tag AP‐MS. (D) The Circos plot was drawn to visualize gene associations with the top 10 GO molecular function terms. (E) The Circos plot shows the gene associations with significantly enriched KEGG pathways. Co‐IP—co‐immunoprecipitation; ESCC—esophageal squamous cell carcinoma; GO—Gene Ontology; MS—mass spectrometry.

Next, proteins that could interact with *KDM4A‐AS1*‐encoded peptide were identified by Co‐IP assay and MS analysis. The schematic diagram of Co‐IP was presented in Fig. 5A. The sliver staining image of gels after Co‐IP analysis was shown in Fig. 5B. The information on MS‐identified proteins that could interact with *KDM4A‐AS1*‐encoded peptide was shown in Table S7. Also, the common nonspecific proteins in the flag tag AP‐MS were downloaded from the CRAPome database. Combined with MS‐identified proteins in the IgG or Flag group and CRAPome database‐identified nonspecific proteins in the flag tag AP‐MS, 103 proteins were identified to be specific in the Flag group after removing the proteins in the IgG group and proteins identified in the CRAPome database. GO and KEGG enrichment analysis for the 103 specific proteins in the Flag group showed that these proteins were significantly enriched in terms related to the oxidation–reduction process and fatty acid metabolism (Fig. 5D,E).

### 3.7 Effect of KDM4A‐AS1‐encoded peptide on the expression of fatty acid metabolism‐related genes and redox process in ESCC cells.

**Fig. 6:**
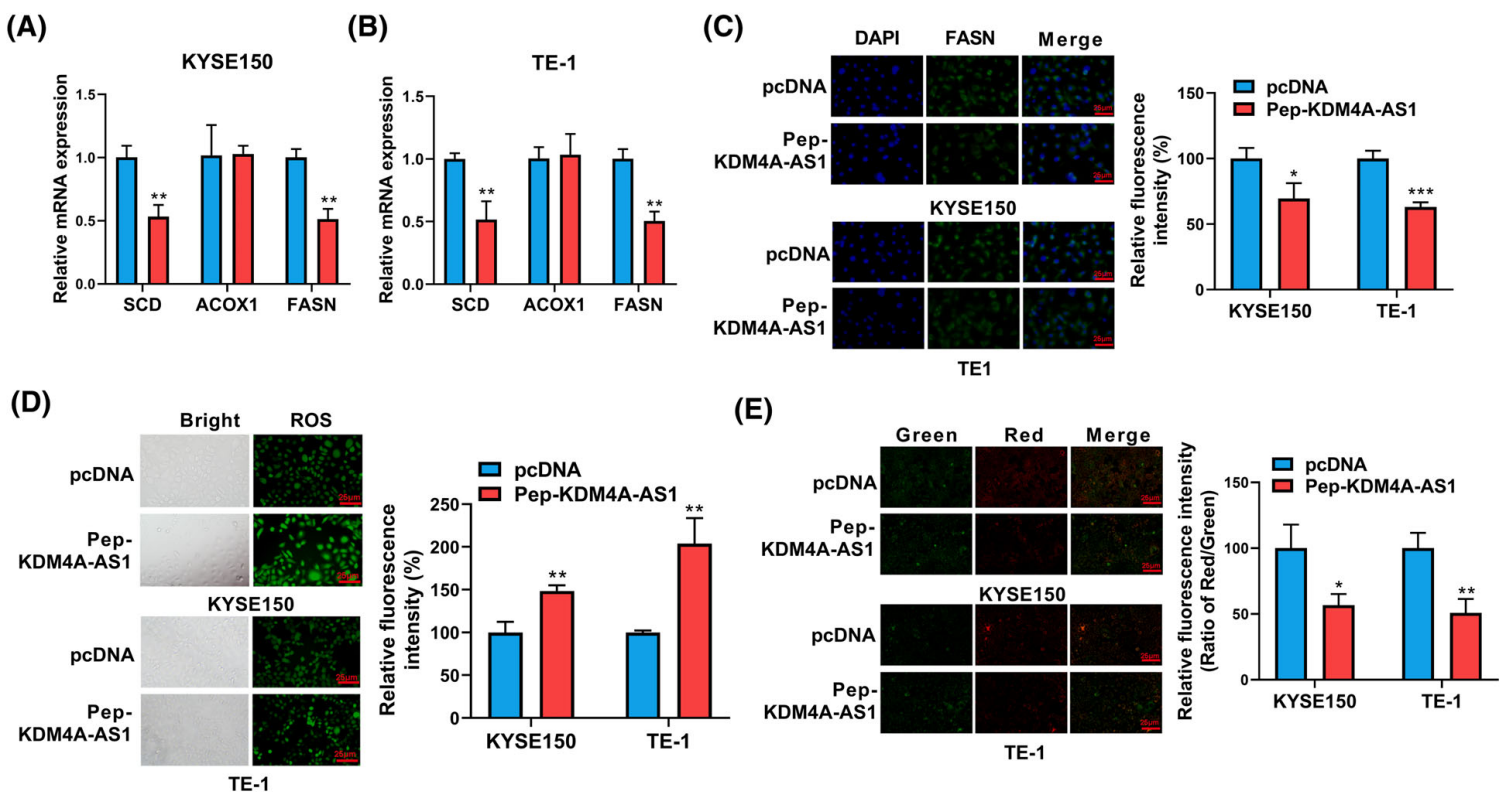
Effect of *KDM4A‐AS1*‐encoded peptide on the expression of fatty acid metabolism‐related genes and redox process in ESCC cells. (A, B) KYSE150 and TE‐1 cells were transfected with pcDNA3.1 or Pep‐KDM4A‐AS1. At 48 h after transduction, the mRNA levels of SCD, ACOX1, and FASN were measured by RT‐qPCR assay (*n* = 3). (C) KYSE150 and TE‐1 cells were transfected with pcDNA3.1 or Pep‐KDM4A‐AS1. At 48 h after transduction, the IF assay was performed to measure the level of FASN (*n* = 3). Scale bar, 25 μm. (D) KYSE150 and TE‐1 cells were transfected with pcDNA3.1 or Pep‐KDM4A‐AS1. At 48 h after transduction, the ROS level was measured by ROS Assay Kit (*n* = 3). Scale bar, 25 μm. (E) KYSE150 and TE‐1 cells were transfected with pcDNA3.1 or Pep‐KDM4A‐AS1. At 48 h after transduction, MMP was measured by mitochondrial membrane potential assay kit with JC‐1 (*n* = 3). Red fluorescence was detected when MMP was higher. Green fluorescence was detected when MMP was lower. Scale bar, 25 μm. ACOX1—peroxisomal acyl‐coenzyme A oxidase 1; ESCC—esophageal squamous cell carcinoma; FASN—fatty acid synthase; IF—immunofluorescence; MMP—mitochondrial membrane potential; Pep‐KDM4A‐AS1—pcDNA3.1‐ORF3‐flag plasmid; ROS—reactive oxygen species; SCD—stearoyl‐CoA desaturase. The data were analyzed by the Student's *t*‐test, and results were shown as mean ± SD from three independent repeats (*n* = 3). *P < 0.05; **P < 0.01; and ***P < 0.001 vs. pcDNA control group.

Next, RT‐qPCR assay demonstrated that *KDM4A‐AS1*‐encoded peptide could markedly inhibit the expression of stearoyl‐CoA desaturase (SCD) and fatty acid synthase (FASN) (two fatty acid metabolism‐related genes) but did not influence the expression of peroxisomal acyl‐coenzyme A oxidase 1 (ACOX1) in KYSE150 and TE‐1 cells (Fig. 6A,B). IF assay also showed that the fluorescence intensity of FASN was notably reduced in KYSE150 and TE‐1 cells overexpressing *KDM4A‐AS1*‐encoded peptide (Fig. 6C). Moreover, we demonstrated that the overexpression of *KDM4A‐AS1*‐encoded peptide led to an about 1.5‐ or 2.0‐fold increase in the levels of reactive oxygen species in KYSE150 and TE‐1 cells, respectively (Fig. 6D). Additionally, the ectopic expression of *KDM4A‐AS1*‐encoded peptide triggered an approximately 43.3% or 49.1% reduction in MMP in the KYSE150 and TE‐1 cells, respectively (Fig. 6E).

### 3.8 Effect of lncRNA *KDM4A‐AS1* knockdown on ESCC viability and migration

RT‐qPCR assay validated the expression of *KDM4A‐AS1* in KYSE150 and TE‐1 cells (Fig. S2A). Moreover, we demonstrated that the introduction of *KDM4A‐AS1*‐1 shRNA1 led to an approximately 40% reduction in the viability of KYSE150 or TE‐1 cells (Fig. S2B). The viability of KYSE150 or TE‐1 cells reduced by about 50% after the introduction of *KDM4A‐AS1* shRNA2 (Fig. S2B). Transwell migration assay revealed that *KDM4A‐AS1* loss led to about 35% reduction in the migrated number of KYSE150 or TE‐1 cells (Fig. S2C). Wound healing assay showed that *KDM4A‐AS1* knockdown triggered an approximately 25% decrease in the migration area of KYSE150 or TE‐1 cells, respectively (Fig. S2D).

## 4 Discussion

Although bioinformatics prediction analysis and high‐throughput technologies suggest some lncRNAs have potential peptide‐ or protein‐coding abilities, a limited number of lncRNAs‐encoded peptides have been validated and the roles of few lncRNAs‐encoded peptides in carcinoma progression have been explored [30, 31]. Also, prior studies have mainly focused on the investigations of the peptide‐coding potential of certain specific interested lncRNAs along with peptide‐related functions [32, 33]. Few studies have been performed to predict and identify lncRNAs with peptide‐coding potential on a large scale by combining multiple methods such as ORF prediction, IRES prediction, ribosome immunoprecipitation, ribosome profiling, and MS identification.

In this study, WGCNA analysis was performed to identify genes and lncRNAs related to ESCC histological grade and TNM stage, which might contribute to the understanding of ESCC metastasis and explore genes or lncRNAs related to ESCC progression. Also, lncRNAs that could bind with ribosomes were identified based on prior ribosome footprint and sequencing datasets. Ribosome footprint and sequencing analysis can disclose the small ORFs within lncRNAs that could interact with ribosomes at the genomic scale and present the potential translation ability of ORFs by the deep sequencing of ribosome‐bound fragments [34, 35]. Next, potential ORFs of lncRNAs associated with both ESCC progression and ribosome translation were predicted. Moreover, peptides in ESCC were identified by comparing the ORFs‐translated peptides against the ESCC MS dataset. Among MS‐identified peptides, the coding potential of 12 peptides‐matched lncRNA ORFs was examined by western blotting assay. In summary, we demonstrated that 6 lncRNAs (*LINC01116*, *KDM4A‐AS1*, *KMT2E‐AS1*, *LINC00839*, *UBL7‐AS1*, *DLX6‐AS1*) related to ESCC progression could encode short peptides in ESCC by the combined analysis of WGCNA, prior ribosome‐seq data, ORF prediction, MS identification, and western blotting assay. Moreover, our data showed that the small peptide that was encoded by *KDM4A‐AS1* could notably weaken ESCC cell viability and migratory ability.

IRESs have been identified as RNA regulatory elements that play vital roles in recruiting ribosomes, mediating ribosomal assembly, and initiating protein translation [36, 37]. TRAP is a technology that can be used to capture and isolate ribosome‐associated RNAs including lncRNAs, quantify translating RNA, and study translational regulation [38, 39, 40]. TransLnc database showed that there was an IRES element on the Pep‐KDM4A‐AS1‐corresponding RNA sequence and *KDM4A‐AS1* could interact with ribosomes. TRAP assay demonstrated that ribosomal protein RPL10A could bind with lncRNA *KDM4A‐AS1*, suggesting the translation potential of *KDM4A‐AS1*. Due to the deficiency of antibodies against Pep‐KDM4A‐AS1 peptide, TRAP assay, and ESCC MS dataset identification are very imperative to validate the translation potential of lncRNA *KDM4A‐AS1* in ESCC. Moreover, ribosomes containing RPL10A could regulate translation by IRES element [41], further suggesting the translation potential of *KDM4A‐AS1*. Additionally, the mutation of the putative *KDM4A‐AS1* ORF start codon abrogated the translation ability of putative *KDM4A‐AS1* ORF in KYSE150 and TE‐1 cells, further validating the peptide‐encoding capacity of putative lncRNA *KDM4A‐AS1* ORF. Furthermore, the *KDM4A‐AS1*‐encoded peptide had stronger hydrophilia. It has been reported that the interfaces of carcinoma‐related proteins are more hydrophilic relative to noncarcinoma proteins [42], further suggesting the vital role of a *KDM4A‐AS1*‐encoded peptide in carcinomas.

Co‐IP and MS analysis suggested that 103 proteins could specifically bind with *KDM4A‐AS1*‐encoded peptide in ESCC. GO and KEGG enrichment analyses for the 103 proteins that could specifically bind with *KDM4A‐AS1*‐encoded peptide showed that these proteins were significantly enriched in terms related to the oxidation–reduction process and fatty acid metabolism. Fatty acids play essential roles in multiple aspects of carcinomas such as energy production, cell proliferation, membrane formation, and signaling transduction [43]. The reprogramming of fatty acid metabolism is closely associated with carcinoma malignant progression and metastasis [44, 45]. Moreover, the dysregulation of fatty acid metabolism has been identified to be closely linked with ESCC progression including ESCC cell migration [46, 47]. Among genes related to fatty acid metabolism, we further investigated the effect of *KDM4A‐AS1*‐encoded peptide on SCD and FASN expression in ESCC cells. Results showed that the overexpression of *KDM4A‐AS1*‐encoded peptide could inhibit the expression of SCD and FASN in KYSE150 and TE‐1 cells. It has been reported that the inhibition of SCD presented antitumor activity in ESCC [48, 49]. Also, some studies demonstrated that FASN loss notably hindered ESCC cell proliferation and migration [46, 50].

The oxidation–reduction (redox) process also plays a vital role in cancer cell metastasis, migration, and invasion [51, 52]. Redox‐related proteins can regulate ROS sensing and metabolism [53]. Also, MMP has been reported to be implicated in ROS production [54]. Our present study demonstrated that the overexpression of *KDM4A‐AS1*‐encoded peptide increased the ROS level and reduced MMP in ESCC cells.

## 5 Conclusions

Taken together, we demonstrated that 6 lncRNAs (*LINC01116*, *KDM4A‐AS1*, *KMT2E‐AS1*, *LINC00839*, *UBL7‐AS1*, and *DLX6‐AS1*) had peptide‐coding potential. Moreover, *KDM4A‐AS1*‐encoded peptide could reduce cell viability and inhibit cell migration in ESCC. This peptide could exert its functions by influencing fatty acid metabolism and the redox process. The identification of novel functional peptides might contribute to the better management of ESCC given their advantages in clinical practices such as physiological noninvasiveness, easily‐absorption, well‐metabolism, and lower antigenicity [55]. To our knowledge, this is the first study that simultaneously identifies and validates a batch of lncRNAs with peptide‐coding potential by combining bioinformatics, transcriptomics, proteomics, and cellular/molecular experiments, which might provide new identification and experimental strategies for peptides that are derived from initially deemed noncoding RNAs.

## Conflict of interest

The authors declare no conflict of interest.

## Author contributions

BZ provided the conceptualization, performed the experiments, wrote the manuscript, and acquired funding support. YW contributed to the data analysis and image visualization. PC and CW contributed to the performance of experiments.

## Supporting information


**Fig. S1.** The identification flowchart of lncRNAs with potential translation abilities in ESCC
**Fig. S2.** Effects of lncRNA *KDM4A‐AS1* knockdown on ESCC cell viability and migration

**Table S1.** The integrated information on 12 filtered peptides and corresponding ORFs and lncRNAs, including differential expression patterns of lncRNAs in 77 TCGA ESCC samples versus 11 normal samples, sequences of peptides and ORFs, and WGCNA modules
**Table S2.** The MS searching parameters against the ESCC MS dataset IPX0002962000
**Table S3.** The information on putative peptides that were encoded by predicted ORFs of lncRNAs that were related to histological grades in ESCC in the WGCNA analysis and ribosome binding in the SRR1758391 and SRR8550319 datasets
**Table S4.** Mass spectrometry‐identified peptide fragments that might be encoded by lncRNAs that were related to histological grades in ESCC in the WGCNA analysis and ribosome binding in the SRR1758391 and SRR8550319 datasets
**Table S5.** The information on putative peptides that were encoded by predicted ORFs of lncRNAs that were related to TNM stages in ESCC in the WGCNA analysis and ribosome binding in the SRR1758391 and SRR8550319 datasets
**Table S6.** Mass spectrometry‐identified peptide fragments that might be encoded by lncRNAs that were related to TNM stages in ESCC in the WGCNA analysis and ribosome binding in the SRR1758391 and SRR8550319 datasets
**Table S7.** The information on MS‐identified proteins that could interact with KDM4A‐AS1‐encoded peptide

## Data Availability

The data that support the findings of this study were shown in supplementary materials. The mass spectrometry proteomics data have been deposited to the iProX partner repository with the dataset identifier PXD038125 (https://www.iprox.cn//page/SCV017.html?query=IPX0005406000).
