# Supplementary material for: Long noncoding RNAs with peptide‐encoding potential identified in esophageal squamous cell carcinoma: KDM4A‐AS1‐encoded peptide weakens cancer cell viability and migratory capacity
Source: Mol Oncol. 2023 Apr 10;17(7):1419–36. doi: 10.1002/1878-0261.13424 (PMC10323902; doi:10.1002/1878-0261.13424)
Supplement: Supplementary file 1 — Fig. S1. The identification flowchart of lncRNAs with potential translation abilities in ESCC Fig. S2. Effects of lncRNA KDM4A‐AS1 knockdown on ESCC cell viability and migration [file MOL2-17-1419-s001.docx]

**Supplementary Figures**

**
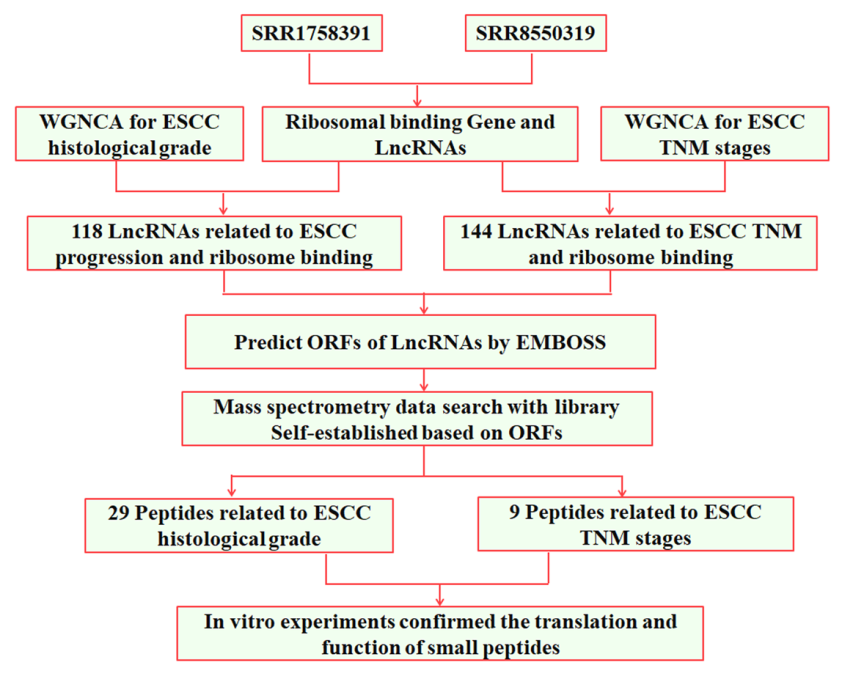
**

**Supplementary Fig. 1 The identification flowchart of lncRNAs with potential translation abilities in ESCC.** ESCC: esophageal squamous cell carcinoma.

**
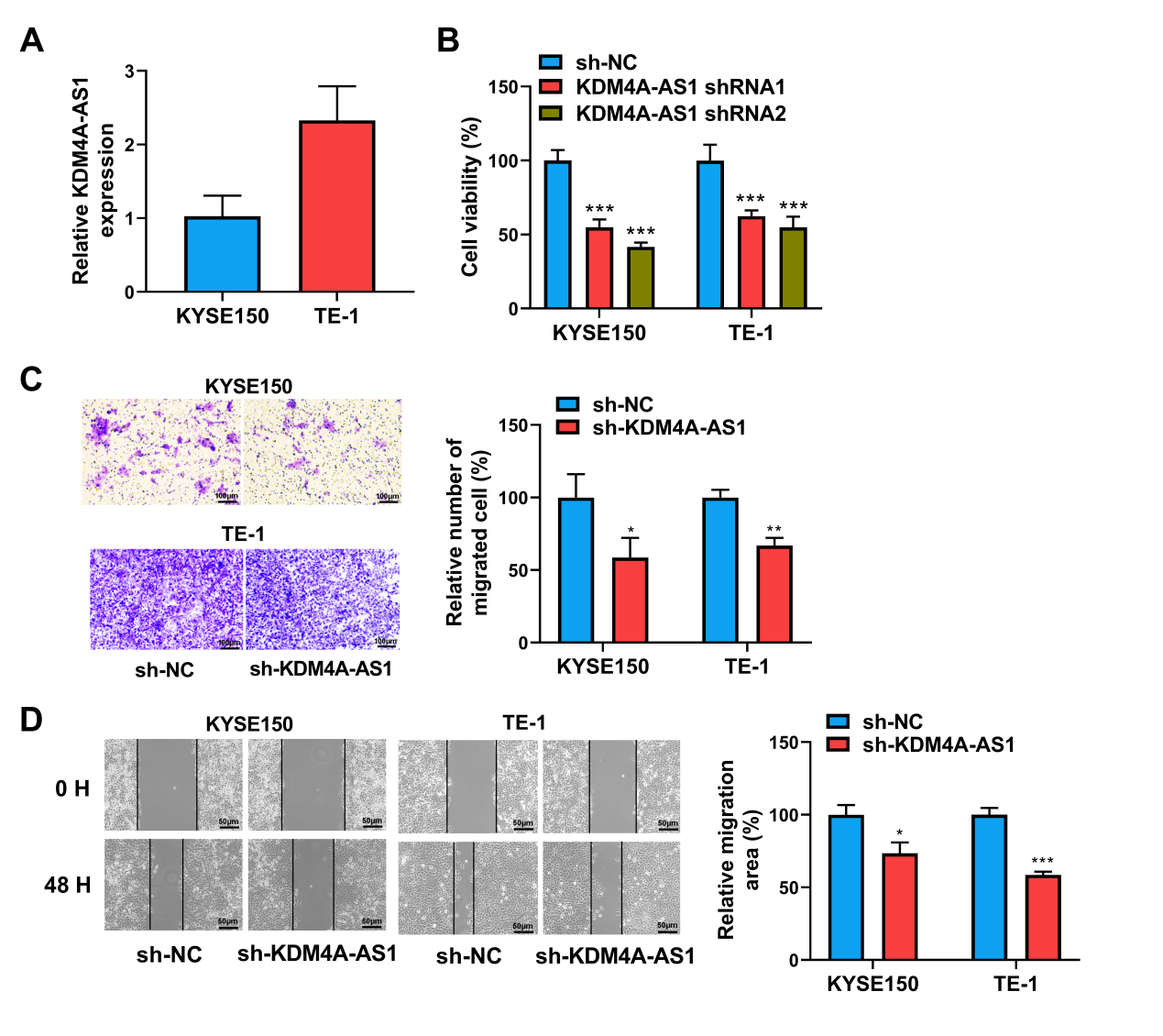
**

**Supplementary Fig. 2 Effects of lncRNA KDM4A-AS1 knockdown on ESCC cell viability and migration.** (A) KDM4A-AS1 level was measured by RT-qPCR assay in TE-1 or KYSE150 cells. (B) TE-1 or KYSE150 cells were infected with sh-NC, KDM4A-AS1 shRNA1, or KDM4A-AS1 shRNA2. At 48 h after transduction, cell viability was examined by CCK-8 assay. The data were analyzed by ANOVA and Tukey test and the results were presented as mean ± standard deviation from 3 independent repeats. (C) TE-1 or KYSE150 cells transduced with sh-NC or sh-KDM4A-AS1 were plated into the upper chambers of transwell plates. At 48 h after transfection, migrated cell number was calculated. The data were analyzed by Student's t-test and results were shown as mean ± standard deviation from 3 independent repeats (n=3). (D) TE-1 or KYSE150 cells were transduced with sh-NC or sh-KDM4A-AS1 (KDM4A-AS1 shRNA2). When cell confluency reached approximately 100%, the wounds were created. The wound regions were imaged at 0 h and 48 h after scratching. The data were analyzed by Student's t-test and results were shown as mean ± standard deviation from 3 independent repeats (n=3). **P* < 0.05; ***P* < 0.01; and ****P* < 0.001 vs. sh-NC control group. ESCC: esophageal squamous cell carcinoma. CCK-8: Cell Counting Kit-8.
